# DNA Damage Response Regulation Alleviates Neuroinflammation in a Mouse Model of α-Synucleinopathy

**DOI:** 10.3390/biom15070907

**Published:** 2025-06-20

**Authors:** Sazzad Khan, Himanshi Singh, Jianfeng Xiao, Mohammad Moshahid Khan

**Affiliations:** 1 Department of Neurology, College of Medicine, University of Tennessee Health Science Center, Memphis, TN 38163, USA; mkhan61@uthsc.edu (S.K.); hsingh10@uthsc.edu (H.S.); jxiao@uthsc.edu (J.X.); 2 Center for Muscle, Metabolism and Neuropathology, Division of Regenerative and Rehabilitation Sciences and Department of Physical Therapy, College of Health Professions, University of Tennessee Health Science Center, Memphis, TN 38163, USA; 3 Neuroscience Institute, University of Tennessee Health Science Center, Memphis, TN 38163, USA

**Keywords:** Parkinson’s disease, AZD1390, DNA damage, α-synuclein, behavioral functions, inflammation, senescence

## Abstract

Parkinson’s disease (PD) is a progressive neurodegenerative disorder marked by the degeneration of dopaminergic neurons in the substantia nigra, leading to decreased dopamine levels in the striatum and causing a range of motor and non-motor impairments. Although the molecular mechanisms driving PD progression remain incompletely understood, emerging evidence suggests that the buildup of nuclear DNA damage, especially DNA double-strand breaks (DDSBs), plays a key role in contributing neurodegeneration, promoting senescence and neuroinflammation. Despite the pathogenic role for DDSB in neurodegenerative disease, targeting DNA repair mechanisms in PD is largely unexplored as a therapeutic approach. Ataxia telangiectasia mutated (ATM), a key kinase in the DNA damage response (DDR), plays a crucial role in neurodegeneration. In this study, we evaluated the therapeutic potential of AZD1390, a highly selective and brain-penetrant ATM inhibitor, in reducing neuroinflammation and improving behavioral outcomes in a mouse model of α-synucleinopathy. Four-month-old C57BL/6J mice were unilaterally injected with either an empty AAV1/2 vector (control) or AAV1/2 expressing human A53T α-synuclein to the substantia nigra, followed by daily AZD1390 treatment for six weeks. In AZD1390-treated α-synuclein mice, we observed a significant reduction in the protein level of γ-H2AX, a DDSB marker, along with downregulation of senescence-associated markers, such as p53, Cdkn1a, and NF-κB, suggesting improved genomic integrity and attenuation of cellular senescence, indicating enhanced genomic stability and reduced cellular aging. AZD1390 also significantly dampened neuroinflammatory responses, evidenced by decreased expression of key pro-inflammatory cytokines and chemokines. Interestingly, mice treated with AZD1390 showed significant improvements in behavioral asymmetry and motor deficits, indicating functional recovery. Overall, these results suggest that targeting the DDR via ATM inhibition reduces genotoxic stress, suppresses neuroinflammation, and improves behavioral outcomes in a mouse model of α-synucleinopathy. These findings underscore the therapeutic potential of DDR modulation in PD and related synucleinopathy.

## 1. Introduction

Parkinson’s disease (PD) is a common neurodegenerative disorder marked by the loss of midbrain dopaminergic neurons, leading to motor symptoms such as tremors, rigidity, and bradykinesia, along with non-motor features, like sleep disturbances and mood changes. PD greatly affects quality of life and places emotional and financial strain on patients, families, and healthcare systems. Currently, over 10 million people worldwide have PD, with projections predicting 25.2 million by 2050, a 112% increase since 2021 [1]. Despite ongoing research, no treatments exist to slow or halt progression, highlighting the urgent need for new therapeutic strategies as the disease’s incidence rises with aging populations. DNA damage in neurodegeneration and proteinopathies is long established [2,3,4,5,6]. Emerging evidence implicates DNA damage, specifically nuclear DNA double-strand breaks (DDSBs), in aging and multiple neurodegenerative diseases, including PD [3,7,8,9]. This vulnerability is expected, as neurons are highly metabolically active and post-mitotic, making them prone to accumulating genomic instability. Despite these insights, targeting DNA repair pathways as a therapeutic strategy in PD remains largely unexplored.

When DNA damage occurs, cells launch the DNA damage response (DDR), a complex network of pathways that detect the damage, transmit signals, and augment repair. The ataxia telangiectasia mutated (ATM) kinase plays a key role in coordinating DDR signaling to promote DNA repair [10,11]. Upon DDSB, ATM undergoes autophosphorylation and triggers the recruitment of a multiprotein repair complex including phosphorylated H2A.X and MRN complex (MRE11-RAD50-NBS1) to facilitate the DNA repair [11,12,13]. If DDSBs are left unrepaired, aberrant DDR signaling can lead immune responses and cellular senescence, both of which are strongly linked to neurodegenerative conditions [5,14]. Persistent ATM activation has been observed in mouse models of PD and other neurodegenerative diseases, exacerbating neuronal damage [15,16,17,18,19]. Inhibiting ATM has shown neuroprotective potential across different disease models. For instance, Lu et al. demonstrated that suppressing ATM, either genetically or pharmacologically, alleviated mutant Huntingtin toxicity both in vitro and in vivo [17]. KU-55933, a selective ATM inhibitor, has demonstrated neuroprotection against MPP^+^-induced neuronal apoptosis [18]. Similarly, KU-55933-mediated ATM inhibition suppresses etoposide-induced DNA damage and apoptosis in resting human T cells [20]. Additionally, caffeine, a non-specific ATM inhibitor, has been found to be a neuroprotector in experimental models [21,22]. Pathogenic variants in LRRK2, the most prevalent genetic contributor to familial PD, interact with ATM to regulate the Mdm2-p53 axis in response to genotoxic stress [23]. DNA damage promotes LRRK2 phosphorylation, which is abolished by ATM deficiency, highlighting ATM as a critical regulator of LRRK2 signaling. These findings suggest that ATM inhibition could offer a promising therapeutic approach by modulating genotoxic stress in neurodegenerative diseases, such as PD. However, the inability of existing ATM inhibitors to cross the blood–brain barrier (BBB) limits their use in the disease course.

AZD1390 is a highly selective, BBB-penetrant ATM kinase inhibitor initially developed to enhance radiosensitivity in tumors [24]. Emerging evidence suggests that ATM inhibition with AZD1390 may have profound effects on brain function. For examples, AZD1390 promotes neurite growth, supports axon regeneration and improves functional outcomes in experimental models of spinal cord injury [25]. AZD1390 has demonstrated efficacy in reducing neuroinflammation and brain damage in an experimental model of ischemic stroke [26,27]. The pharmacokinetic and pharmacodynamic profiles as well as clinical efficacy of AZD1390 have been recently documented in clinical studies of brain tumors [24,28]. In the present study, we demonstrate that ATM inhibition with AZD1390 significantly reduces neuroinflammatory and senescence markers and improves behavioral function in the context of α-synuclein-induced neurotoxicity. These results establish DDR modulation as a promising therapeutic strategy for PD and related synucleinopathy.

## 2. Materials and Methods

### 2.1. Cell Cultures

SH-SY5Y cells (ATCC, Manassas, VA, USA) were cultured in Dulbecco’s Modified Eagle Medium/Nutrient Mixture F-12 (Corning, NY, USA; #10-090-CV) composed of 10% fetal bovine serum (FBS) and 1% penicillin-streptomycin. Upon reaching confluence, cells were treated with 10 µM etoposide for 6 h. A separate group of cells received etoposide treatment followed by incubation with increasing concentrations (0, 5, 10, and 20 nM) of the ATM inhibitor AZD1390 for 24 h. Cell viability was assessed using the MTT assay according to the standard protocol (ATCC; 30-1010K). Primary cortical neurons were prepared from embryonic day 14 (E14) wild-type (WT) mouse embryos, as previously described [9,29]. Briefly, cortices were dissected and enzymatically dissociated, and the resulting cells were counted and plated onto poly-L-lysine-coated coverslips in 24-well plates. Neurons were cultured in Neurobasal plus medium (Thermo Fisher Scientific; #A35829010; Waltham, MA USA) supplemented with 2% B27 (Thermo Fisher Scientific; A3582801), 1% FBS, and 1 mM L-glutamine. Cultures were incubated at 37 °C with 5% CO_2_, and the medium was refreshed every three days. Neurons were cultured for 14 days in vitro (DIV14) before treatments. To induce DNA damage, neurons were treated with etoposide (10 µM) for 6 h, with or without co-treatment with AZD1390 (10 nM). Following etoposide exposure, AZD1390-treated cultures were maintained for an additional 24 h before fixation and analysis.

### 2.2. Mice

C57BL/6J (WT) mice were purchased from The Jackson Laboratory, Bar Harbor, ME, USA (Strain # 000664). The mouse experiments were conducted in compliance with the National Institutes of Health’s Guidelines for the Care and Use of Laboratory Animals and received approval from our Institutional Animal Care and Use Committee (IACUC). To assess the impact of ATM inhibition on DNA damage and neuroinflammation in the synucleinopathy mouse model, WT mice were divided into three experimental groups. Given estrogen’s well-established neuroprotective effects against various neurotoxic insults, we conducted all experiments using male mice to eliminate potential confounding influences of endogenous female hormones on the interpretation of AZD1390’s efficacy in a PD model. In Group 1, mice were injected unilaterally with an empty AAV1/2 vector and served as controls. Group 2 mice received a unilateral injection of an AAV1/2 vector carrying the mutated A53T α-synuclein gene into the substantia nigra (SN), modeling the disease. In Group 3, synucleinopathy mice were treated daily with the ATM inhibitor AZD1390 (20 mg/kg, orally: Cat. No.: HY-109566; MedChem Express, Monmouth Junction, NJ, USA) for 6 weeks. The AZD1390 dose was chosen based on prior studies demonstrating its neuroprotective effects in animal models [25,26].

### 2.3. Mouse Model of α-Synucleinopathy

A mouse model of α-synucleinopathy was developed as previously described [30,31]. Briefly, male WT mice (4-month-old; C57BL/6J) were anaesthetized with isoflurane (1.5–2%) and mounted on a stereotaxic frame. The skin over the skull was incised to expose the bregma, and the coordinates of the SN were precisely measured as AP −3.1 mm, lateral −1.4 mm, and dorsoventral −4.2 mm relative to bregma as described previously [32]. A small burr hole was drilled over the SN on one side of mouse brain and a unilateral injection was performed with either an empty AAV1/2 vector (control) or an AAV1/2 vector carrying the mutated A53T form of the α-synuclein gene (AAV1/2-CMV/CBA-human-A53T-alpha-synuclein-WPRE-BGH-polyA; Charles River Laboratories, Wilmington, MA, USA). In each instance, a 2 µL injection of the viral vector (5.1 × 10^12^ genomic particles (gp)/mL) was delivered to the SN at a rate of 0.5 µL/min using a Hamilton syringe and programmable Harvard pump system. Following injection, the needle was left in place for an additional 5 min before being gradually removed. Six weeks after stereotaxic injections, mice were assessed for behavioral function and euthanized for morphological and biochemical analysis.

### 2.4. Behavioral and Motor Function Assessments

All behavioral assays including the cylinder test, CatWalk test, and raised beam task tests were performed as described previously [33,34,35,36,37].

### 2.5. Cylinder Test

Spontaneous forepaw use was evaluated six weeks after AAV1/2 injection using the cylinder test. Mice were placed in a transparent plexiglass cylinder and video recorded for 5 min. A blinded observer scored the number of wall contacts made using the ipsilateral, contralateral, or both forelimbs. The proportion of contacts made with the ipsilateral (right) forepaw was calculated as a percentage of total contacts and reported as ipsilateral forelimb use.

### 2.6. Raised-Beam Task

Motor coordination and balance were evaluated using a beam-walking task in which mice were trained to cross an 80-cm-long, 20-mm-wide beam elevated 50 cm above a cushioned surface. To encourage movement, a 60 W light source was placed at the starting end as an aversive stimulus, while a darkened escape box was positioned at the other end of the beam. The time taken to cross the beam and the number of paw slips were recorded. Following initial testing on a 20-mm square beam, additional trials were conducted using 9-mm round and square beams. The mean values were used for statistical analysis.

### 2.7. CatWalk Gait Analysis

Gait assessments were performed six weeks post-AAV1/2 injection using the CatWalk automated gait analysis system (Noldus Information Technology, Wageningen, The Netherlands). This video-based system evaluates voluntary walking by capturing footstep patterns as mice traverse a glass plate runway illuminated with a fluorescent light. A high-speed camera positioned below the plate recorded the movements, and CatWalk XT 10.0 software was used for analysis. Key gait parameters, including walking duration, and average speed were assessed. Each mouse completed three trials, and mean values were used for further analysis.

### 2.8. Relative Quantitative Real-Time Reverse-Transcriptase PCR (RT-qPCR)

Relative mRNA levels in mouse tissues were quantified using SYBR Green-based RT-qPCR on Roche’s LightCycler^®^ 480 System (Indianapolis, IN, USA), following previously established protocols [9,34]. Total RNA was isolated from homogenized midbrain tissues using TRI Reagent^®^ (Thermo Fisher Scientific, Waltham, MA, USA). cDNA synthesis was performed using random primers in accordance with the RETROscript™ Reverse Transcription Kit protocol (Thermo Fisher Scientific). Gapdh served as the endogenous control. Relative gene expression changes were calculated using the 2^−ΔΔCT^ comparative method and presented as a fold change. Primer sequences are provided in the Supplementary Material (Table S1).

### 2.9. Immunocytochemistry

To assess DDSB, cells were fixed with 4% paraformaldehyde (PFA) for 15 min at room temperature and processed for immunocytochemistry. Following fixation, cells were permeabilized with 0.1% Triton X-100 in PBS for 10 min and subsequently blocked with 5% BSA in PBS for 1 h at room temperature. Neurons were then incubated overnight at 4 °C with the anti-γH2AX (Ser139) antibody (1:500; BioLegend, San Diego, CA, USA) to detect DDSBs and the anti-MAP2 antibody (EnCor Biotechnology Inc., Gainesville, FL, USA) to label neuronal cells. After washing, cells were incubated with fluorescently labeled secondary antibodies, followed by DAPI nuclear staining. Images were captured using a fluorescence microscope, and γH2AX foci per nucleus were quantified to assess DNA damage levels. The details of the antibodies used are summarized in the Supplementary Material (Table S2).

### 2.10. Immunofluorescence Staining in Mouse Brain

Immunostaining was carried out as described previously by us [9,38]. In brief, mice were anesthetized with isoflurane, and their brains were swiftly harvested and immersed in a 4% paraformaldehyde solution. The brains were sectioned at 20 μm using a cryostat. Following PBS washes, sections were blocked with 5% BSA in PBS for 1 h at room temperature. Primary antibodies were then applied, and sections were incubated overnight at 4 °C with primary antibodies, including anti-TH (BioLegend or EMD Millipore; Massachusetts, MA, USA), anti-HA tag (Cell Signaling, Danvers, MA, USA), or phospho-ATM (Ser1981) (Invitrogen; Carlsbad, CA, USA). Detection was carried out using secondary antibodies conjugated to Alexa Fluor 488 or Alexa Fluor 555. After three additional PBS washes, sections were counterstained with DAPI, and images were acquired using a fluorescence microscope.

### 2.11. Western Blot Analyses

Midbrain tissue was extracted from each mouse and lysed in ice-cold RIPA buffer (Thermo Fisher Scientific, USA) supplemented with Halt™ protease and phosphatase inhibitor cocktail (Thermo Fisher Scientific, USA). The lysates were centrifuged at 12,000 rpm for 20 min, and the resulting supernatants were collected for protein analysis. Proteins were separated using SDS-PAGE on 4–20% Criterion™ TGX™ Precast Midi Protein Gels (Bio-Rad, Hercules, CA, USA) and transferred onto PVDF membranes using a wet transfer system. Membranes were blocked with 5% BSA for 1 h before overnight incubation with primary antibodies, including mouse γ-H2AX (Ser139), rabbit phospho NF-κB, rabbit phospho-p53, and rabbit GAPDH (10494-1-AP; Proteintech, Rosemont, IL, USA), prepared in antibody diluting solution. After washing with TBST, membranes were incubated with HRP-conjugated secondary antibodies for 2 h at room temperature (goat anti-rabbit #A9169 or rabbit anti-mouse #A9044; Millipore Sigma, Burlington, MA, USA) while continuously rocking. Protein detection was performed using the SuperSignal™ West Pico or Femto ECL kit (Thermo Fisher Scientific, USA), and membranes were imaged using the Odyssey Fc imaging system (Li-Cor, Lincoln, NE, USA). Protein band intensities were analyzed with NIH ImageJ software (1.54g; Bethesda, MD, USA), and expression levels were normalized to GAPDH as a loading control. Raw images of Western blots were provided in Supplementary Materials.

### 2.12. Statistical Analysis

All statistical analyses were carried out using GraphPad Prism 10.2 (GraphPad Software, San Diego, CA, USA). Data were tested for normality using the Shapiro–Wilk test. For parametric data, one-way ANOVA followed by appropriate post hoc tests (with correction for multiple comparisons against a baseline control group) was used to evaluate treatment effects. The Kruskal–Wallis test was applied to assess non-parametric behavioral measures, such as slips in the raised beam task. Statistical significance was set at *p* < 0.05, and results are expressed as the mean ± SEM.

## 3. Results

### 3.1. AZD1390 Mitigates DNA Damage in Etoposide-Exposed Neuronal Cultures and in the Brains of α-Synucleinopathy Mice

**Figure 1:**
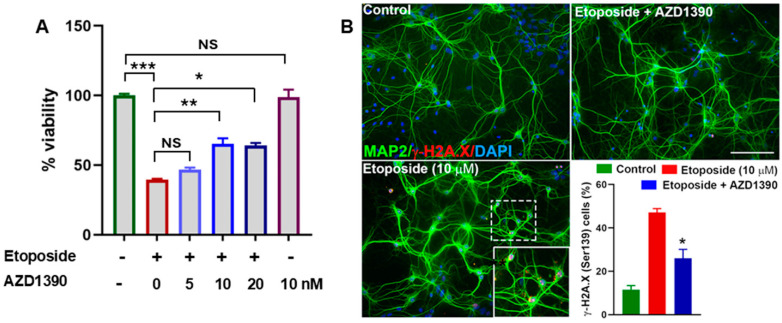
ATM inhibition improves cell viability and reduces etoposide-induced DNA damage in cell cultures. (**A**) Effect of AZD1390 on SH-SY5Y cell viability following etoposide-induced DNA damage. SH-SY5Y cells were treated with increasing concentrations of the ATM inhibitor AZD1390 (0, 5, 10, and 20 nM) in the presence of etoposide. Cell viability was assessed using the MTT assay. Data are presented as the mean ± SEM from three independent experiments. Data were compared between groups using one-way ANOVA. * *p* < 0.05, ** *p* < 0.01, AZD1390 vs. etoposide; *** *p* < 0.001 etoposide vs. control. NS = non-significant. (**B**) Representative immunofluorescence images of mouse neuronal cultures showing the colocalization of DDSB marker [γ-H2AX (Ser139); red] and neuronal marker (MAP2; green). Quantification of γ-H2AX foci in MAP2-positive neurons was performed to assess DDSB accumulation. The solid box shows a magnified view of the area marked by the white dashed box in the main image. Data were compared between groups using one-way ANOVA and are presented as the mean ± SEM * *p*< 0.05; AZD1390 vs. etoposide. Scale bar = 100 µM; magnification = 200×.

**Figure 2:**
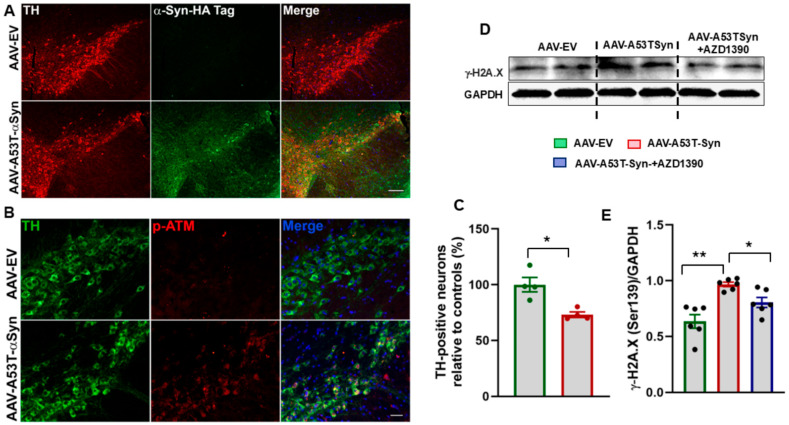
ATM inhibition reduces DNA damage in the midbrain of a mouse model of α-synucleinopathy. (**A**) Representative immunofluorescence images showing α-synuclein (HA-Tag; green) localized in dopaminergic neurons labeled with tyrosine hydroxylase (TH; red) in the substantia nigra (SN), six weeks after AAV1/2-A53T-α-Syn vector injection into the WT mouse brain. (N = 4/group). Magnification 100×; Scale bar, 200 μm. (**B**) Immunofluorescence staining for phospho-ATM (p-ATM; red) in midbrain sections of WT mice infused with AAV1/2-A53T-αSyn vector. Elevated p-ATM signal was observed in dopaminergic neurons, identified by co-labeling with tyrosine hydroxylase (TH; green), indicating activation of the DNA damage response in α-synuclein-expressing neurons. Scale bar, 50 μm. (N = 4/group). (**C**) quantification of TH-positive neurons. (**D**,**E**) Immunoblot analysis of γ-H2A.X (Ser139) levels in the midbrain of AAV-EV and AAV-A53T-Syn-treated WT and AAV-A53T-Syn-treated WT followed by AZD1390 treatment (N = 6 mice/group). Data were compared between groups using one-way ANOVA with a Holm–Sidak post hoc test. The values are represented as mean ± SEM * *p* < 0.05; ** *p* < 0.01.

We first evaluated the effect of ATM inhibitor AZD1390 on cell viability in SH-SY5Y cells exposed to topoisomerase II inhibitor etoposide, a DNA damaging agent, using the MTT assay across a range of concentrations (0, 5, 10, and 20 nM). Treatment with AZD1390 at 10 nM resulted in a significant increase in cell viability compared to the untreated control (0 nM), indicating a potential pro-survival or cell protective effect (Figure 1A). Importantly, no overt cytotoxicity was observed at this concentration. Lower concentrations (5 nM) elicited a modest incraese in viability, while a slight reduction in viability was noted at 20 nM. These findings suggest that AZD1390 enhances cell viability at an optimal concentration (10 nM). To assess whether AZD 1390 reduces DDSB accumulation, mouse primary cortical neurons were exposed to etoposide for 6 hr. Our data demonstrated that etoposide treatment led to a pronounced induction of DDSBs in neurons, as evidenced by a significant increase in γ-H2A.X foci formation. This robust DNA damage response was indicative of substantial genotoxic stress induced by etoposide. However, when neurons were co-treated with AZD1390, we observed a significant reduction in γ-H2AX foci, suggesting that ATM inhibition effectively mitigated etoposide-induced DNA damage (Figure 1B). We then extended our study to assess the impact of AZD1390 in a mouse model of α-synucleinopathy. We first examined A53T α-synuclein protein expression in the SN and found it to be predominantly localized within dopaminergic neurons (Figure 2A). This expression was associated with significant loss of dopaminergic neurons (Figure 2C) and pronounced synuclein pathology, as indicated by elevated levels of Ser129-phosphorylated α-synuclein (Figure S2). Notably, we also observed a marked upregulation of ATM phosphorylation in these neurons following α-synuclein accumulation (Figure 2B), consistent with previous reports linking ATM activation to synuclein pathology [15]. To determine whether ATM activation was associated with genotoxic stress, we quantified γ-H2A.X expression in the midbrain of α-synuclein-expressing WT mice, with or without ATM inhibitor AZD1390 treatment. AZD1390 at a dose of 20 mg/kg has been previously shown to significantly reduce phosphorylated ATM levels in the central nervous system. Based on this evidence, we selected this dose for our study [25]. Moreover, AZD1390 treatment was well tolerated, with no observable adverse effects or significant changes in body weight throughout the study (Figure S1). Our analysis revealed a significant increase in γ-H2A.X expression in the midbrain of α-synuclein-treated mice compared to vehicle-treated WT controls, indicating heightened DNA damage in response to α-synuclein pathology. Notably, treatment with AZD1390 significantly (F_2,15_ = 13.42; *p* = 0.0005) reduced γ-H2A.X levels in α-synuclein-treated mice, suggesting that ATM inhibition effectively mitigates DNA damage (Figure 2C,D), reinforcing the hypothesis that the reduction in DNA damage, as indicated by lower γH2A.X levels, is likely a consequence of ATM suppression.

### 3.2. ATM Inhibition Suppresses Senescence in α-Synuclein-Treated Mice

**Figure 3:**
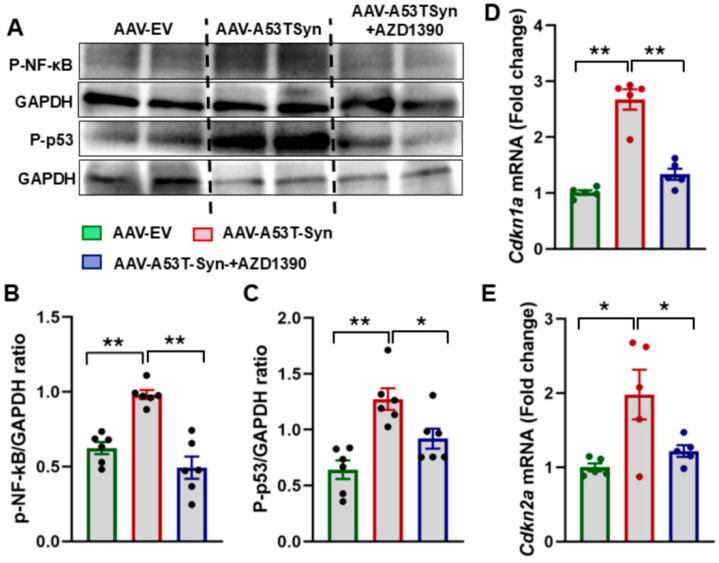
AZD1390 reduces senescence in the brains of α-synucleinopathy mice. (**A**–**C**) Western blot analysis was conducted to assess phosphorylated p53 (P-p53) and phosphorylated NF-κB (P-NF-κB) in midbrain tissue lysates from AAV-EV control, α-synuclein-expressing, and α-synuclein + AZD1390-treated WT mice. Band intensities were quantified by densitometry and normalized to GAPDH. Statistical significance was determined using one-way ANOVA followed by Holm–Sidak post hoc test. Data are presented as the mean ± SEM (N = 6/group). * *p* < 0.05, ** *p* < 0.01. (**D**,**E**) The mRNA expression of senescence markers *Cdkn1a* and *Cdkn2a* assessed by RT-qPCR. Data were analyzed using one-way ANOVA followed by a Holm–Sidak post hoc test; * *p* < 0.05 or ** *p* < 0.01 was considered statistically significant (N = 5/group).

Sustained DNA damage is a key driver of cellular senescence, a state in which cells permanently cease to divide and often adopt a pro-inflammatory profile referred to as the senescence-associated secretory phenotype (SASP). Given that ATM is a critical regulator of the DDR and senescence pathways, we investigated whether its inhibition via AZD1390 could suppress senescence in α-synuclein-treated mice. Our analysis revealed a significant upregulation of *Cdkn1a* and *Cdkn2a* mRNA levels in α-synuclein-treated mice compared to WT controls, reinforcing the idea that α-synuclein accumulation promotes a senescent phenotype in midbrain cells. However, AZD1390 treatment markedly reduced the expression of both genes, suggesting that ATM inhibition effectively suppresses senescence-associated transcriptional activation (Figure 3D,E). To further assess senescence, we evaluated the expression levels of phospho-p53 (p-p53), and phospho-NF-κB (pNF-κB) in midbrain tissues. α-Synuclein-treated mice showed a significant increase in these markers compared to WT controls. Notably, AZD1390 treatment significantly reduced the expression levels of p-p53 (F_2,15_ = 12.38; *p* = 0.0007), and pNF-κB (F_2,15_ = 23.90; *p* < 0.0001), demonstrating that ATM inhibition mitigates DNA damage-induced senescence (Figure 3A–C).

### 3.3. AZD1390 Reduces Neuroinflammatory Responses in the Brains of α-Synuclein-Treated Mice

**Figure 4:**
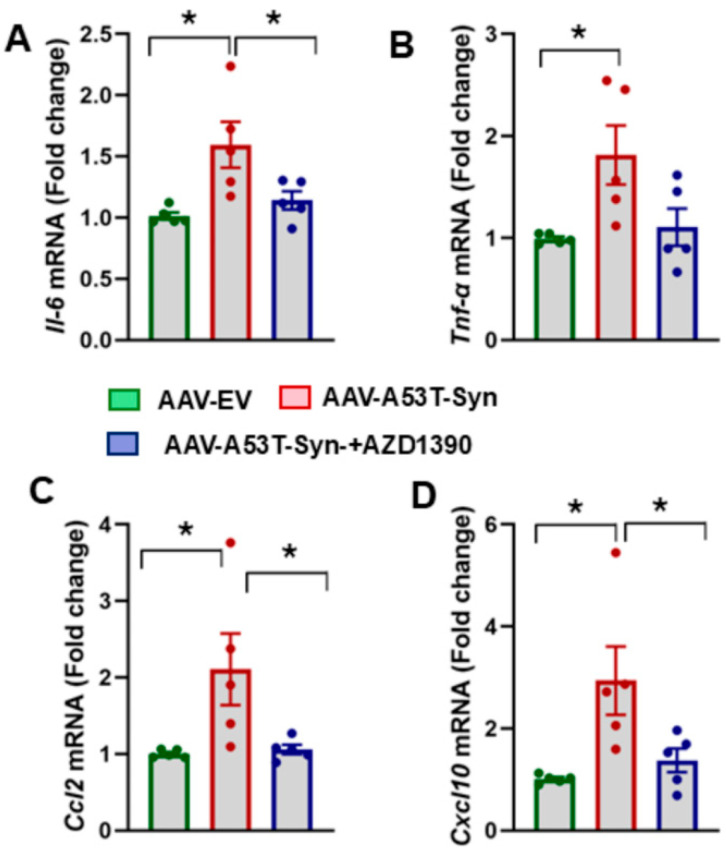
AZD1390 reduces neuroinflammatory responses in the brains of α-synucleinopathy mice. RT-qPCR as employed to assess the mRNA expressions of cytokines *Il-6 *(**A**) and *Tnf*-α (**B**) and chemokines *Ccl2 *(**C**), and *Cxcl10 *(**D**) in midbrains of controls, AAV-A53T-Syn-treated mice and AAV-A53T-Syn-injected mice treated with AZD1390 post 6 weeks of vector infusion. Data were analyzed using one-way ANOVA followed by the Holm–Sidak post hoc test. The values are expressed as the mean ± SEM * *p* < 0.05 (N = 5/group).

Neuroinflammation plays a central role in PD, with pro-inflammatory cytokines and chemokines contributing significantly to the progression of neurodegeneration. Given the link between ATM activation and inflammatory signaling, we investigated whether AZD1390 could attenuate the expression of key pro-inflammatory cytokines/chemokines in α-synuclein-treated mice. Our analysis revealed a significant increase in the mRNA expression levels of cytokines *Tnf-α* and *Il-6* in the midbrain of α-synuclein-treated mice compared to controls. Notably, AZD1390 treatment reduced the expression of these cytokines, suggesting that ATM inhibition suppresses neuroinflammation in α-synucleinopathy (Figure 4A,B). To further assess the neuroinflammatory responses, we quantified the mRNA expression levels of chemokines *Ccl2* and *Cxcl10*, which are known to recruit immune cells and sustain neuroinflammation in PD. Our results showed that *Ccl2* and *Cxcl10* mRNA levels were significantly upregulated in the midbrain of α-synuclein-treated mice compared to controls, indicating a dysregulated chemokine response. Notably, there was a significant effect of AZD1390 treatment on the mRNA expression levels of *Ccl2* (F_2,12_ = 5.187; *p* = 0.0238) and *Cxcl10* (F_2,12_ = 6.253; *p* = 0.0138) in α-synuclein-treated mice (Figure 4C,D), suggesting that ATM inhibition suppresses neuroinflammatory responses.

### 3.4. AZD1390 Improves Behavioral Function in a Mouse Model of α-Synucleinopathy

**Figure 5:**
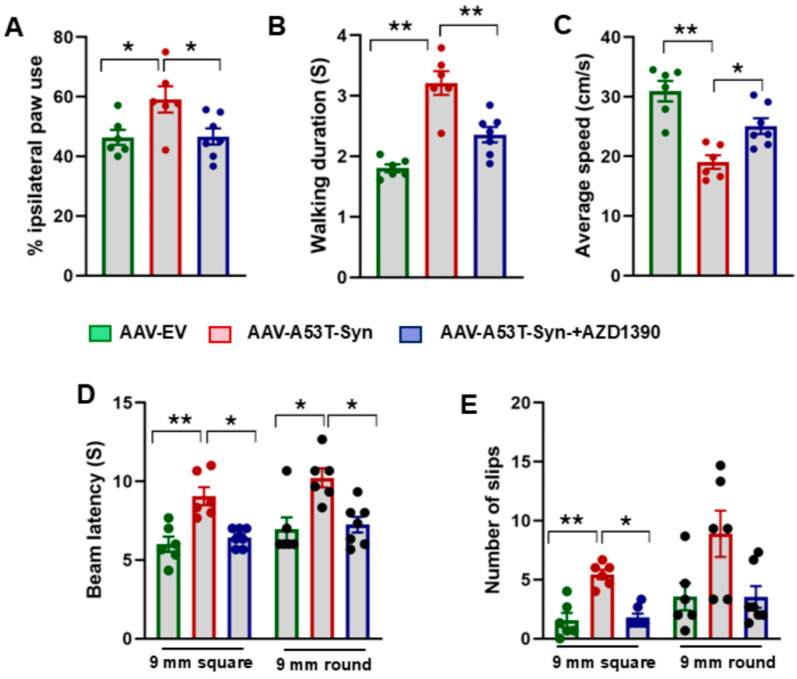
Inhibition of ATM alleviates behavioral deficits in a mouse model of α-synucleinopathy. (**A**) The cylinder test was performed to evaluate forelimb asymmetry in AAV-A53T-αSyn-expressing mice, with or without treatment with the AZD1390. The frequency of contact with the cylinder wall using the impaired versus non-impaired forelimb was quantified to assess motor asymmetry. Data were analyzed using one-way ANOVA followed by the Holm–Sidak post hoc test. The values are expressed as the mean ± SEM * *p* < 0.05. (**B**,**C**) Gait analysis was conducted using the CatWalk system to evaluate walking duration and average speed in α-synuclein-expressing mice, with or without AZD1390 treatment. Data were analyzed using one-way ANOVA followed by the Holm–Sidak post hoc test. The values are expressed as the mean ± SEM * *p* < 0.05; ** *p* < 0.01. (**D**,**E**) Raised beam task was used to assess the ability of mice to walk on narrow transverse beam to enter a dork box and number of slips during walking on 9 mm square and round beams. Motor performance was assessed by measuring beam latency and counting the number of slips during traversal. Beam latency data were analyzed using one-way ANOVA, while the number of slips, a non-parametric behavioral measure, was assessed using the Kruskal–Wallis test. The values are expressed as mean ± SEM * *p* < 0.05; ** *p* < 0.01. N = 6–7 mice/group.

To assess whether AZD1390 treatment improves motor coordination, balance, and locomotion, we conducted a series of well-established behavioral tests (Figure 5A–E). In the cylinder test, α-synuclein-treated mice displayed reduced spontaneous use of the contralateral forelimb, a hallmark of unilateral dopamine depletion. There was a significant effect of AZD1390 treatment (F_2,16_ = 4.782; *p* = 0.0236) on forelimb symmetry, likely due to an improvement in striatal dopamine function (Figure 5A). In CatWalk gait analysis, α-synuclein-treated mice displayed abnormal gait parameters, including reduced walking duration and decreased average speed. AZD1390 treatment significantly restored gait parameters, suggesting improved locomotor function and sensorimotor coordination (Figure 5B,C). In the raised beam task, α-synuclein-treated mice had difficulty traversing the beam, with increased slips and longer traversal times. AZD1390 significantly improved balance and precision in movement, with fewer slips and shorter traversal times (Figure 5D,E). One-way analysis showed a significant effect of treatment on beam latency for both the square (F_2,16_ = 13.78; *p* = 0.0003) and round (F_2,16_ = 8.286; *p* = 0.0034) 9 mm beams. Similarly, treatment had a significant effect on the number of slips on square (*p* < 0.01) and round beam (*p* < 0.05) as determined by the Kruskal–Wallis test. Our findings demonstrate that AZD1390 significantly improves motor and behavioral function while preventing neuroinflammation in a mouse model of α-synucleinopathy.

## 4. Discussion

Parkinson’s disease is characterized by progressive dopaminergic neurodegeneration, α-synuclein accumulation, and chronic neuroinflammation, all of which contribute to disease progression. Strong experimental evidence demonstrates that sustained accumulation of DNA damage triggers multiple signaling pathways involved in the pathophysiology of age-related neurodegenerative diseases. Increased DNA damage has been observed in human PD brains and experimental models of PD and related synucleinopathies. Despite this evidence, targeting the DDR has not yet been established as a therapeutic strategy for PD. ATM, a key regulator of DDR, has been implicated in neurodegeneration, and recent studies suggest that its inhibition may offer therapeutic benefits. Our study demonstrates that AZD1390, a potent ATM inhibitor, exerts beneficial effects by reducing genotoxic stress (DNA damage), suppressing senescence and neuroinflammation, and improving behavioral function in a mouse model of α-synucleinopathy.

ATM inhibition may exert its protective effects in PD by mitigating genotoxic stress possibly arising from oxidative damage and other cellular insults common in neurodegenerative settings. Another potential mechanism is through its impact on nuclear transcription and chromatin remodeling. Given that ATM activation induces widespread chromatin modifications and gene silencing at DNA damage sites [39], its inhibition could modulate these processes in ways that influence PD progression. Notably, several chromatin-modifying genes regulated by ATM have been implicated in α-synuclein interactions [40,41,42], suggesting a possible link between ATM signaling and synuclein pathology. However, the precise contributions of transcriptional regulation and chromatin remodeling to ATM-mediated neuroprotection in PD remain unclear. Additionally, ATM inhibition may influence disease progression by suppressing senescence-associated signaling pathways. ATM activation, while protective in the short term and plays a protective role under physiological conditions, can become maladaptive if sustained, contributing to excessive genomic instability, neuronal dysfunction, and cell death, especially in the context of neurodegenerative diseases.

In this study, we demonstrate that ATM inhibition via AZD1390 effectively dampens the excessive activation of the DDR, thereby reducing DNA damage accumulation and enhancing neuronal resilience against etoposide-induced toxicity. These findings align with previous reports indicating that ATM inhibition attenuates etoposide-mediated DNA damage and apoptosis by modulating DDR signaling [20,22]. Expanding our investigation into an α-synucleinopathy mouse model, we assessed the impact of ATM inhibition on genotoxic stress, a key driver of neurodegeneration in PD. Given that pathogenic α-synuclein triggers genotoxic stress by activating ATM and γ-H2A.X [15,43], we sought to determine whether ATM blockade could mitigate these effects. Our results reveal that α-synuclein-overexpressing mice exhibit heightened ATM and γ-H2A.X expression, indicative of sustained DNA damage and an overactive DDR. Strikingly, treatment with AZD1390 significantly reduces γ-H2A.X levels, underscoring its ability to limit DNA damage accumulation and prevent excessive DDR activation. This is consistent with prior studies demonstrating that ATM inhibition mitigates DNA damage and neuronal loss in neurodegenerative models [17,44,45]. These findings reinforce the notion that dysregulated ATM activation exacerbates genomic instability and neuronal stress, both of which are pivotal in the pathophysiology of PD. Importantly, by inhibiting ATM, we not only mitigate acute genotoxic stress but may also confer long-term protection, potentially disrupting the cascade of secondary neurodegeneration.

ATM serves as a key regulator of senescence induction in response to DNA damage [46,47]. Senescent cells acquire a pro-inflammatory SASP, which amplifies neuroinflammation and accelerates disease progression. Our study reveals that α-synuclein-treated mice exhibit significant upregulation of senescence markers, including phospho-p53 and phospho-NF-κB, alongside increased *Cdkn1a* and *Cdkn2a* mRNA expression, hallmarks of a senescent phenotype. Notably, AZD1390 treatment markedly reduces the expression of these markers, indicating that ATM inhibition effectively suppresses DNA damage-induced senescence. This finding is in line with previous studies demonstrating that ATM inhibition mitigates senescence and SASP in models of aging and age-related diseases [46,48,49,50]. Chronic neuroinflammation is a major contributor to dopaminergic neurodegeneration, characterized by excessive production of pro-inflammatory cytokines and chemokines [51]. ATM has been implicated in NF-κB activation, linking DDR to inflammatory signaling [52]. Our results reveal that α-synuclein-treated mice exhibit heightened expression of pro-inflammatory chemokines (*Ccl2* and *Cxcl10*) and cytokines (*Tnf*-α and *Il-6*). Strikingly, AZD1390 treatment significantly suppresses these inflammatory mediators, indicating that ATM inhibition exerts an anti-inflammatory effect. This aligns with studies demonstrating that ATM inhibition reduces NF-κB-driven inflammation and neurotoxicity [27,46]. Given that increased CCL2 and CXCL10 expression has been reported in PD brains and cerebrospinal fluid [51,53], our findings suggest that ATM inhibition may modulate inflammatory signaling pathways to mitigate neuroinflammation. By targeting ATM, we not only prevent DNA damage accumulation but also disrupt a pathological cycle wherein senescence and inflammation perpetuate functional deficits.

AZD1390 not only exerts profound molecular benefits but also significantly ameliorates behavioral deficits in α-synuclein-treated mice. Motor impairments, such as gait disturbances, balance deficits, and forelimb asymmetry, are hallmark features of α-synucleinopathy, yet treatment with AZD1390 leads to marked improvements in motor coordination, balance, and exploratory behavior, suggesting notable functional recovery. Our study has several limitations that warrant further investigation. Although our findings suggest that ATM inhibition reduces genotoxic stress, senescence, and inflammation, the precise molecular mechanisms connecting these processes remain to be fully elucidated. Additionally, given ATM’s critical role in maintaining genomic stability across various tissues, the long-term inhibition of ATM by AZD1390 could potentially disrupt immune function or impair tumor suppression, thereby increasing cancer risk. Thus, careful modulation of ATM activity is essential to balance its neuroprotective benefits, such as reducing senescence and curbing neuroinflammation, with the need to preserve genomic integrity. While our results demonstrate significant attenuation of molecular pathology and improvement in behavioral outcomes following AZD1390 treatment, a key limitation of the study is the absence of direct assessment of dopaminergic neuron survival or nigrostriatal pathway integrity. Although the observed effects align with a potential therapeutic profile, definitive conclusions regarding neuronal preservation cannot be drawn from the current data. In light of this limitation, we emphasize that the neuroprotective effects of AZD1390 remain a hypothesis, warranting future studies with comprehensive analysis of the striatonigral pathway. An important consideration for future studies is the influence of sex and age on AZD1390 efficacy. Parkinson’s disease shows sex-specific differences in incidence and treatment response, shaped by hormonal and genetic factors. Aging also impacts neurodegeneration and DNA repair, potentially affecting the therapeutic window and long-term outcomes of ATM inhibition. Future research should evaluate these variables to determine whether AZD1390’s molecular and behavioral benefits are consistent across different sexes and age groups. These insights will be crucial for optimizing therapies and advancing personalized interventions. Despite these challenges, our results underscore the dual molecular and functional benefits of ATM inhibition, positioning AZD1390 as a promising therapeutic candidate for mitigating the genotoxic stress, neuroinflammation, and motor deficits characteristic of PD.

## 5. Conclusions

In summary, our study demonstrates that ATM is aberrantly activated in a mouse model of α-synucleinopathy, and its pharmacological inhibition attenuates the DNA damage response, reduces neuroinflammation, and improves behavioral performance. These findings underscore ATM as a viable therapeutic target and suggest that safeguarding genomic integrity may help counteract early pathogenic events across neurodegenerative diseases.

## Data Availability

The raw data supporting the conclusions of this article will be made available by the authors on request.

## Supplementary Materials

The following supporting information can be downloaded at: https://www.mdpi.com/article/10.3390/biom15070907/s1, Figure S1: Body weight measurements before and after AZD1390 or vehicle treatment; Figure S2: α-synuclein (Ser129) accumulation in the mouse brain following AAV1/2-A53T-αSyn delivery; Figure S3: Analysis of phosphorylated NF-κB levels in midbrain lysates of treated mice; Table S1: Primers for RT-qPCR in mice; Table S2: Antibodies used in Western blot and immunohistochemistry; File S1: Western Blotting Figures.
