# Supplementary material for: DNA Damage Response Regulation Alleviates Neuroinflammation in a Mouse Model of α-Synucleinopathy
Source: Biomolecules. 2025 Jun 20;15(7):907. doi: 10.3390/biom15070907 (PMC12292858; doi:10.3390/biom15070907)
Supplement: Supplementary file 1 [file biomolecules-15-00907-s001.zip › biomolecules-3656789-supplementary-english.pdf]

Table S1: Primers for RT-qPCR in mice

Primer	Sequence (5'→3')	Locus	Species	Product (bp)
**Ccl2_mF**	gctacaagaggatcaccagcag	NM_011333.3 234 - 255	Mouse	
**Ccl2_mR**	gtctggacccattccttcttgg	NM_011333.3 339 - 318	Mouse	116 (with Ccl2_mF)
**Cdkn1a_mF**	tcgctgtcttgcactctggtgt	NM_007669.5 438 - 459	Mouse	
**Cdkn1a_mR**	ccaatctgcgcttggagtgatag	NM_007669.5 561 - 539	Mouse	124 (with Cdkn1a_mF)
**Cdkn2a_mF**	tgttgaggctagagaggatcttg	NM_009877.2 233 - 255	Mouse	
**Cdkn2a_mR**	cgaatctgcaccgtagttgagc	NM_009877.2 346 - 325	Mouse	114 (with Cdkn2a_mF)
**Cxcl10_mF**	atcatccctgcgagcctatcct	NM_021274.2 223 - 244	Mouse	
**Cxcl10_mR**	gacctttttggctaaacgctttc	NM_021274.2 356 - 333	Mouse	134 (with Cxcl10_mF)
**Gapdh_mF**	catcactgccacccagaagactg	NM_008084.4 606 - 628	Mouse	
**Gapdh_mR**	atgccagtgagcttcccgttcag	NM_008084.4 758 - 736	Mouse	153 (with Gapdh_mF)
**Tnf_mF**	ggtgcctatgtctcagcctctt	NM_013693.3 253 - 274	Mouse	
**Tnf_mR**	gccatagaactgatgagagggag	NM_013693.3 391 - 369	Mouse	139 (with Tnf_mF)
**Il6_mF**	taccacttcacaagtcggaggc	NM_031168.2 216 - 237	Mouse	
**Il6_mR**	ctgcaagtgcatcatcgttgttc	NM_031168.2 331 - 309	Mouse	116 (with Il6_mF)

Table S2: Antibodies used in Western blot and immunohistochemistry

Antibodies used in Western blot				
S/NO	Antibody	Manufacturer & location	Catalog number	Dilution
1	Phospho-NF-κB (Ser536)	Cell Signaling, USA	3033	1:1000
2	Anti-NF-kB p65	Abcam, USA	Ab76302	1:1000
3	γH2A.X (Ser139)	BioLegend, USA	613402	1:500
4	Phospho-p53 (Ser15)	Cell signaling, USA	9284	1:1000
5	GAPDH	ProteinTech, USA	10494-1-AP	1:7000
6	Anti-Mouse IgG Peroxidase antibody	Millipore Sigma, USA	A9169	1:5000
7	Anti-Rabbit IgG Peroxidase antibody	Millipore Sigma, USA	A9044	1:5000
Antibodies used in immunohistochemistry				
S/NO	Antibody	Manufacturer & location	Catalog number	Dilution
1	TH	EMD Millipore, USA	AB152	1:500
2	TH	BioLegend, USA	8180002	1:500
3	Phospho-ATM (Ser1981)	Invitrogen	14-9046-80	1:100
4	Anti-HA tag	Cell signaling, USA	3724	1:1000
5	MAP2	EnCor Biotechnology Inc, USA	CPCA-MAP2	1:1000
6	α-synuclein (Ser129)	BioLegend, USA	BioLegend, USA	1:250
7	Alexa Fluor 488 Goat Anti-Rabbit	Invitrogen, USA	A-21424	1:500
8	Alexa Fluor 555 Goat Anti-Mouse	Invitrogen, USA	A-11008	1:500
9	Alexa Fluor 488 donkey Anti-Mouse	Invitrogen, USA	A-21202	1:500
10	Alexa Fluor 488 Goat Anti-Chicken	Invitrogen, USA	A-11039	1:500

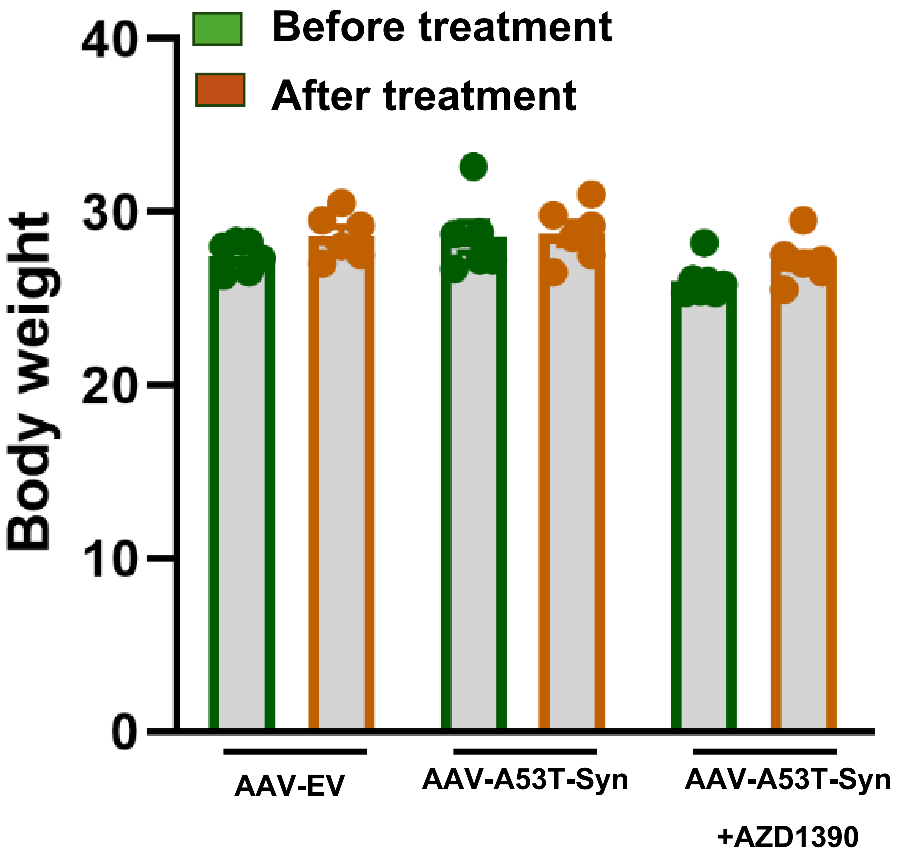

Figure S1. Body weight measurements before and after AZD1390 or vehicle treatment. Body weights of mice from each group were recorded prior to initiation and at the conclusion of AZD1390 treatment. No significant differences in body weight were observed between groups at either time point, indicating that AZD1390 treatment did not affect the overall weight. N=6–7 mice/group.

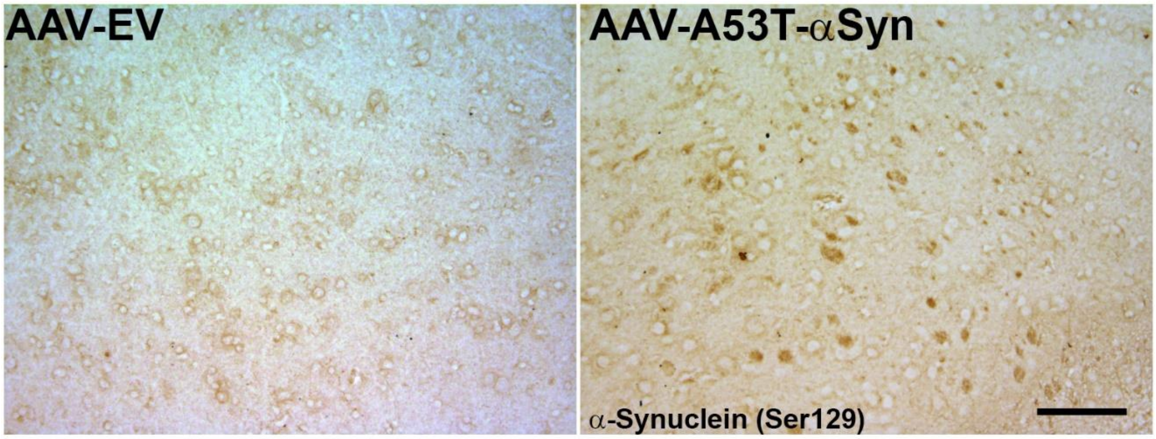

Figure S2. Ser129-phosphorylated α-synuclein accumulation in the mouse brain following AAV1/2-A53T-αSyn delivery. Representative images of Ser129-phosphorylated α-synuclein immunoreactivity in midbrain regions of mice injected with AAV1/2-A53T-αSyn compared to those injected with AAV1/2-empty vector (AAV-EV), indicating pathological α-synuclein accumulation.

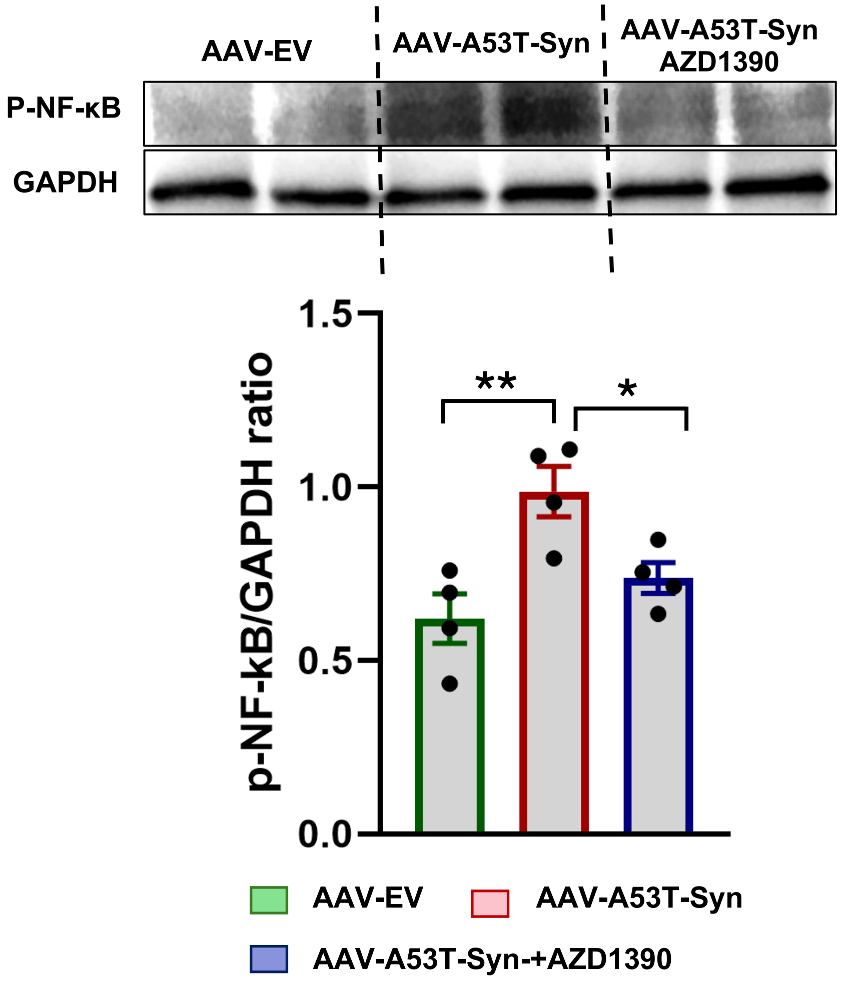

Figure S3. Analysis of phosphorylated NF-κB levels in midbrain lysates of treated mice. Western blotting was performed to evaluate phosphorylated NF-κB (P-NF-κB; Abcam # Ab76302) in midbrain tissue from WT mice receiving AAV-EV (control), AAV1/2-A53T-αSyn, or AAV1/2-A53T-αSyn with AZD1390 treatment. Band intensities were quantified by densitometry and normalized to GAPDH as a loading control. Statistical comparisons were made using one-way ANOVA. Data are expressed as the mean ± SEM (N = 4 per group). *P < 0.05, **P < 0.01.
