# Supplementary figures and images for: DNA Damage Response Regulation Alleviates Neuroinflammation in a Mouse Model of α-Synucleinopathy
Source: Biomolecules. 2025 Jun 20;15(7):907. doi: 10.3390/biom15070907 (PMC12292858; doi:10.3390/biom15070907)

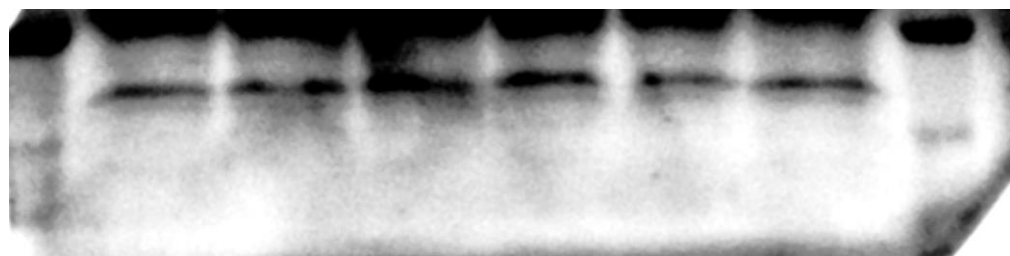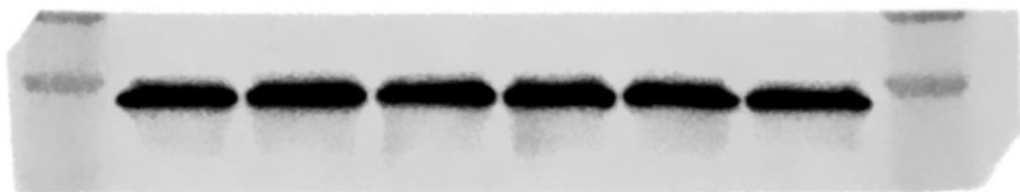

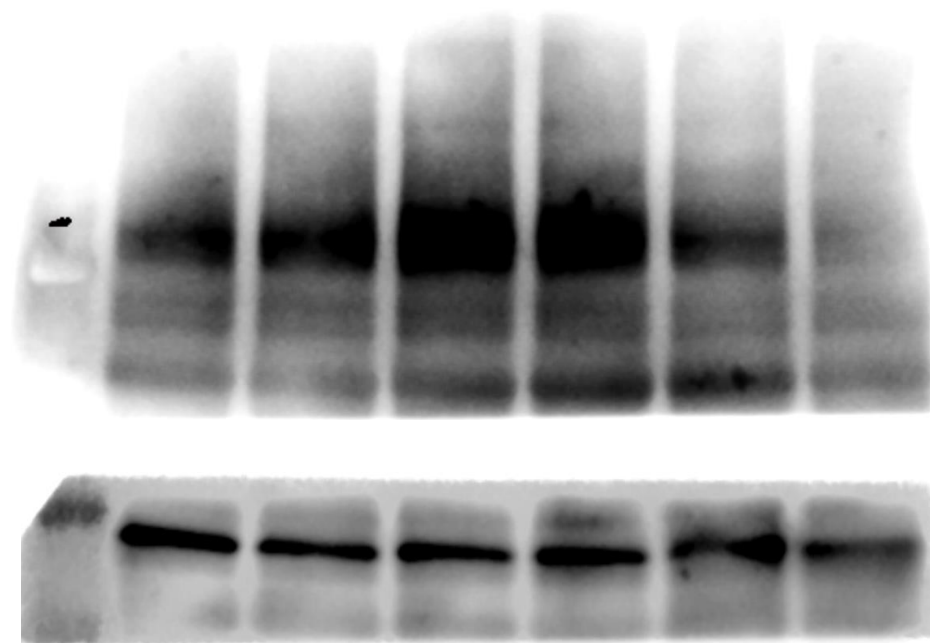

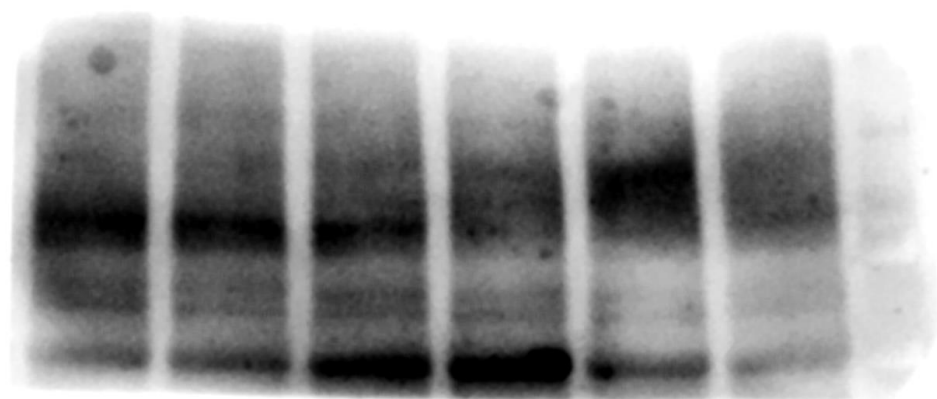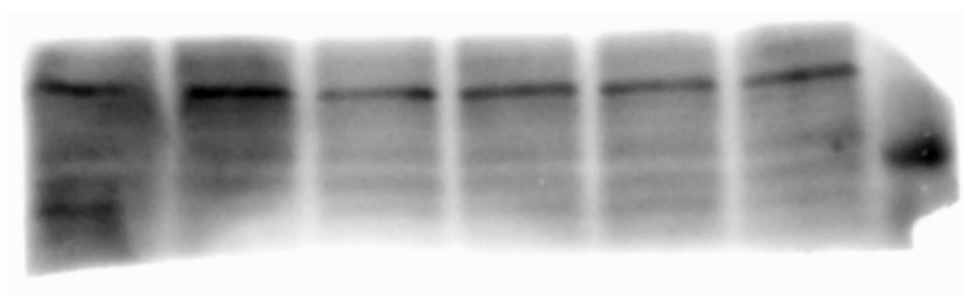

Supplement: Supplementary file 1 [file biomolecules-15-00907-s001.zip › biomolecules-3656789-file S1. original-images.pdf]
